# Serum HER-2 concentration is associated with insulin resistance and decreases after weight loss

**DOI:** 10.1186/1743-7075-7-14

**Published:** 2010-02-25

**Authors:** José Manuel Fernández-Real, Javier A Menendez, Gema Frühbeck, José María Moreno-Navarrete, Alejandro Vazquez-Martín, Wifredo Ricart

**Affiliations:** 1 Department of Diabetes, Endocrinology and Nutrition, CIBERobn Fisiopatología de la Obesidad y Nutrición CB06/03/010, Instituto de Salud Carlos III, 28029 Madrid, Spain; 2 Department of Diabetes, Endocrinology and Nutrition, Girona Biomedical Research Institute (IdIBGi), Hospital Dr Josep Trueta, 17007 Girona, Spain; 3 Department of Oncology, Catalan Institute of Oncology-Girona (ICO-Girona), 17007 Girona, Spain; 4 Department of Endocrinology, Clínica Universitaria de Navarra and CIBERObn Fisiopatología de la Obesidad y Nutrición, Instituto de Salud Carlos III, 31008 Pamplona, Spain

## Abstract

**Background:**

HER2/*neu *is a member of the epidermal growth factor receptor family easily detectable in the serum of cancer patients. We aimed to evaluate circulating HER-2 concentrations in association with insulin resistance in healthy and obese subjects.

**Methods:**

Insulin sensitivity (minimal model) and serum HER-2 concentrations were evaluated in a cross sectional study in men (cohort 1, n = 167) and longitudinally after weight loss in obese subjects (cohort 2, n = 30).

**Results:**

Serum HER-2 concentrations were positively associated with BMI and waist circumference (both r = 0.18, p = 0.02), post-load glucose (r = 0.28, p = 0.001) and fasting triglycerides (r = 0.26, p = 0.001); and negatively associated with insulin sensitivity (r = -0.29, p = 0.002, n = 109). Subjects with type 2 diabetes showed significantly increased soluble serum HER-2 concentrations. In different multivariate regression models, fasting triglycerides emerged as the factor that independently contributed to 10-11% of serum HER-2 variance.

Serum HER-2 concentrations correlated significantly with fasting triglycerides and insulin sensitivity index in subjects from cohort 2. Weight loss led to a significant decrease of serum HER-2 concentrations. The change in serum HER-2 concentrations were significantly associated with the change in percent body fat and fasting triglycerides in young (below the median age of the cohort) subjects.

**Conclusions:**

Serum HER-2 concentrations might be implicated in the pathophysiology of insulin resistance and associated comorbidities.

## Background

Some cancers -including those of the breast [1], colorectum [2], endometrium [3], liver [4], and pancreas [5] occur more commonly in individuals with diabetes. A recent meta-analysis of 20 studies showed that women with (*versus *without) diabetes had a statistically significant 20% increased risk of breast cancer (RR, 1.20; 95% CI, 1.12-1.28) [6].

The mechanisms underlying the relation between type 2 diabetes and breast cancer risk may be related to alterations in circulating concentrations of insulin and insulin-like growth factors (IGFs). It is well known that insulin resistance and increased insulin secretion for long periods are characteristics of type 2 diabetes both before and after disease onset. Insulin has been demonstrated to have mitogenic effects on breast tissue [7,8], and insulin receptors are frequently over-expressed in breast cancer cells [9,10]. A positive association between circulating concentrations of insulin or C-peptide and breast cancer risk has been observed in several [11-15], but not in all epidemiologic studies [16-18]. Elevated insulin concentrations may also stimulate tumor growth by increasing bioavailable IGF-I [19]. High circulating IGF-I concentrations have been shown to predict premenopausal breast cancer risk [20].

The epidermal growth factor receptor (EGFR) family was the first growth factor receptor to be identified in cancer cells [21]. There are four members of the EGFR family: EGFR, *c-erbB-2 *(HER2), erbB3 and erbB4. The unligated, monomeric receptor does not appear capable of signalling. The receptors need to oligomerize -usually in response to ligands- before intracellular signalling is initiated. Each family member has unique characteristics: the EGFR binds ligands and its kinase is activated by asymmetric interactions with the other family members [22].

HER2 (also known as neu and as *c-erbB-2*, which encodes a *M*_r_185,000 transmembrane glycoprotein receptor) is a proto-oncogene of the EGF receptor family of receptor tyrosine kinases that does not bind ligands but is poised to associate with ligand-activated forms of the other family members [22]. These receptors are composed of an extracellular binding domain, a transmembrane lipophilic segment, and an intracellular protein tyrosine kinase domain with a regulatory carboxyl terminal segment [23]. Full-length HER2 undergoes a proteolytic event that results in the release of the soluble ECD/HER-2 fragment and, concomitantly, in the production of an amino-terminally truncated, cell-associated, HER2 fragment that contains the kinase domain (designated as HER2 p95 because of its molecular weight) with enhanced signaling activity [24].

ECD/HER-2 can be released by proteolytic cleavage from the full-length HER2 receptor. Like EGFR, it is a tyrosine kinase receptor whose activation leads to proliferative signals within the cells. Because HER-2 is the preferred dimerization partner when heterodimers are formed, it is important for signaling through ligands specific for any members of the family. Levels of circulating ECD/HER-2 are easily detectable in the serum of breast cancer patients and its measurement has been proved useful to monitor women with metastatic breast cancer, to detect the early appearance of recurrent breast cancer and to predict response to hormonal therapy or chemotherapy [25,26]. Recently, we have found that metformin led to down-regulation of HER-2 expression in *in vitro *models [27]. We hypothesised that the change in insulin action induced by metformin was behind this observation. If this was the case, systemic insulin action might interact with circulating HER-2 concentration. We thus aimed to evaluate circulating HER-2 concentration in association with insulin sensitivity in a cross-sectional study in healthy men, and in a longitudinal study in obese subjects.

## Methods

### Cohort 1 Subjects

One hundred and seventy-seven consecutive men fulfilling inclusion criteria and enrolled in a cross-sectional, population-based study on cardiovascular risk factors in healthy subjects in Northern Spain were studied. All subjects were of Caucasian origin and reported that their body weight had been stable for at least three months before the study. None of the patients were taking any medication or had any evidence of metabolic disease other than obesity. A 75-g oral glucose tolerance test according to the American Diabetes Association Criteria was performed in all subjects. Of these subjects, 100 had strictly normal glucose tolerance, 38 showed impaired glucose tolerance and 29 had previously unknown type 2 diabetes (either in fasting conditions or during the oral glucose tolerance test).

Inclusion criteria for all subjects were: 1) body mass index (BMI) < 40 kg/m^2^, 2) absence of any systemic disease, 3) alcohol intake less than 40 g a day. Informed written consent was obtained after the purpose, nature, and potential risks were explained to the subjects. The protocol was approved by the Hospital Ethics Committee.

### Measurements

Each subject was studied in the research laboratory in the fasting state. Patients were requested to withhold alcohol and caffeine during at least 12 h prior to the insulin sensitivity test. Insulin sensitivity was measured using the frequently sampled intravenous glucose tolerance test in those subjects who agreed (n = 109 of Cohort 1 subjects), as previously described [28].

### Study of the effects of weight loss

Forty-two Caucasian obese volunteers (22 females, 20 males) attending the Endocrinology Department at the University Clinic of Navarra were recruited. Patients underwent a clinical assessment including medical history, physical examination, body composition analysis, co-morbidity evaluation as well as nutritional interviews performed by a multidisciplinary consultation team. All subjects were non-smokers. Patients with signs of infection were excluded. Obese patients were not receiving statins or antidiabetic medication.

Weight loss was achieved by prescription of a diet providing a daily energy deficit of 500-1000 kcal/d as calculated from the determination of the resting energy expenditure through indirect calorimetry (Vmax29, SensorMedics Corporation, Yorba Linda, California) and multiplication by 1.4 as indicated for sedentary individual's to obtain the patient's total energy expenditure. This hypocaloric regime allows a safe and steady weight loss of 0.5-1.0 kg/wk when followed and supplied 30, 54 and 16% of energy requirements in the form of fat, carbohydrates and protein, respectively.

In this study, body weight was measured with a digital scale to the nearest 0.1 kg, and height was measured to the nearest 0.1 cm with a Holtain stadiometer (Holtain Ltd., Crymych, UK). Body fat was estimated by air-displacement-plethysmography (Bod-Pod^®^, Life Measurements, Concord, California, USA). Data for estimation of body fat by this plethysmographic method has been reported to agree closely with the traditional gold standard hydrodensitometry (underwater weighing).

The institutional review board of the participant institutions approved the protocol, so we certify that all applicable institutional regulations concerning the ethical use of information and samples from human volunteers were followed during this research.

### Analytical determinations

The serum glucose concentrations were measured in duplicate by the glucose oxidase method with the use of a Beckman Glucose Analyzer II (Beckman Instruments, Brea, Calif). Total serum cholesterol was measured through the reaction of cholesterol esterase/cholesterol oxidase/peroxidase, using a BM/Hitachi 747. HDL cholesterol was quantified after precipitation with polyethylene glycol at room temperature. Total serum triglycerides were measured through the reaction of glycerol-phosphate-oxidase and peroxidase. HbA1c was measured by the high-performance liquid chromatography method (Bio-Rad, Muenchen, Germany, and autoanalyser Jokoh HS-10, respectively). Intra- and inter-assay coefficients of variation were less than 4% for all these tests.

Serum insulin was measured in duplicate by monoclonal immunoradiometric assay (Medgenix Diagnostics, Fleunes, Belgium). The intra-assay coefficient of variation was 5.2% at a concentration of 10 mU/l and 3.4% at 130 mU/l. The inter-assay coefficients of variation were 6.9% and 4.5% at 14 and 89 mU/l, respectively.

In the *effects of weight loss *study, plasma glucose was analyzed by an automated analyzer (Roche/Hitachi Modular P800) as previously described [29] Insulin was measured by means of an enzyme-amplified chemilumi-nescence assay (IMMULITE^®^, Diagnostic Products Corp., Los Angeles, CA, USA). To estimate insulin resistance, the HOMA index was calculated as fasting insulin concentration (μU/mL) × fasting glucose concentration (mmol/L)/22.5.

#### HER2-specific enzyme-linked immunosorbent assay

Determinations of circulating HER2 concentrations were centralized in a single laboratory and performed with a commercially available quantitative ELISA (Oncogene Science, Bayer Diagnostics) according to the manufacturer's protocol. Intra- and interassays coefficients of variation were lower than 8%. Two controls (high and low, as provided by the manufacturer) were run in each assay to confirm that recoveries fell within the expected ranges (9.8 ± 0.69 and 3.3 ± 0.34 ng/ml, respectively) for each level. Free IGF-I was determined using a 2-site immunoradiometric assay (IRMA) kit (Diagnostic Systems Laboratories; Webster, TX). The detection limit was 0.03 ng/mL, with an intra-assay variation less than 10% for concentrations below 2 ng/mL.

### Statistical methods

Descriptive results of continuous variables are expressed as mean ± SD. Before statistical analysis, normal distribution and homogeneity of the variances were evaluated using Levene's test and then variables were given a log-transformation if necessary. These parameters (triglycerides, insulin sensitivity, and serum HER-2) were analyzed on a log scale and tested for significance on that scale. Relation between variables were tested using Pearson's test and stepwise multiple linear regression analysis. We used chi-square test for comparisons of proportions, and ANOVA test with post-hoc Sheffé's test for comparisons of quantitative variables across categories of glucose tolerance. For a given value of p = 0.05, the study had a 99% power to detect significant correlations between parameters in the whole sample of subjects in a bilateral test (n = 167). The analysis were performed using the program SPSS (version 14.0).

## Results

### Cohort 1 subjects

**Figure 1:**
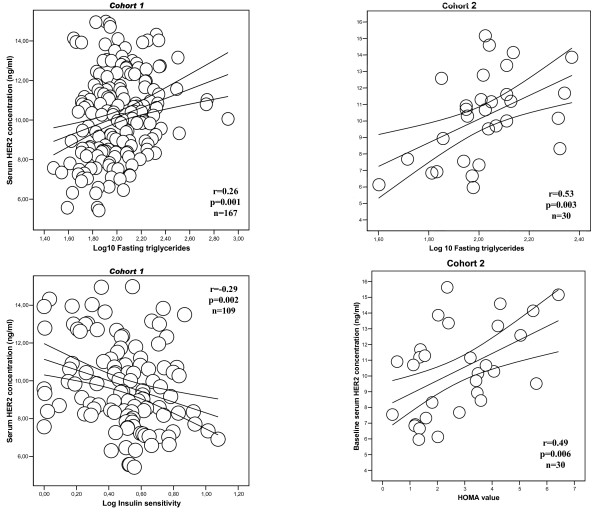
**Linear relationhip between serum HER-2 concentration and log-fasting triglycerides and insulin resistance in subjects from Cohort 1 [left panels] and in subjects from the weight loss study [Cohort 2, right panels]**.

Anthropometric and biochemical characteristics of the Cohort 1 men are shown in Table 1. Serum HER-2 concentrations were positively associated with BMI and waist circumference (both r = 0.18, p = 0.02), post-load glucose (r = 0.28, p = 0.001), glycated hemoglobin (r = 0.16, p = 0.03), total cholesterol (r = 0.23, p = 0.003), LDL-cholesterol (r = 0.20, p = 0.01) and fasting triglycerides (r = 0.26, p = 0.001, Figure 1); and negatively associated with free IGF-I (r = -0.26, p = 0.001) and insulin sensitivity (r = -0.29, p = 0.002, Figure 1). Serum HER-2 concentrations were not associated with age, fasting glucose or HDL-cholesterol (r < 0.12, p > 0.1).

**Table 1:** Clinical and biochemical variables of Cohort 1 subjects

	Normal glucose tolerance	Impaired glucose tolerance	Type 2 diabetes	ANOVA p
**N**	100	38	29	-
**Age [years]**	50.7 ± 13	59.6 ± 10.2	58 ± 12.1	<0.0001
**BMI [kg/m^2^]**	26.8 ± 3.3	28.3 ± 3.4	31.8 ± 6.9	<0.0001
**Waist [cm]**	90.4 ± 8.6	94.3 ± 9.1	104.6 ± 15.3	<0.0001
**Systolic blood pressure [mm Hg]**	124.7 ± 13	132.4 ± 17.3	138.8 ± 16.4	<0.0001
**Diastolic blood pressure [mm Hg]**	79 ± 9.1	83 ± 10.4	84.2 ± 11	0.03
**Cholesterol [mg/dl]**	205.5 ± 40.3	227.4 ± 41.7	211.8 ± 34.8	0.01
**LDL-cholesterol [mg/dl]**	132.8 ± 37.2	149.2 ± 34.6	129.3 ± 37.8	0.03
**HDL- cholesterol [mg/dl]**	53 ± 12.4	51.4 ± 11.5	47.3 ± 12	0.09
**Log-Triglycerides [mg/dl]**	1.94 ± 0.21	2.06 ± 0.21	2.13 ± 0.25	<0.0001
**Fasting glucose [mg/dl]**	95 ± 9.8	102.3 ± 11.5	138.5 ± 23	<0.0001
**Fasting insulin [mU/l]**	8 ± 4	12.1 ± 6.2	14.3 ± 9.7	<0.0001
**HbA_1c _[%]**	4.8 ± 0.55	4.9 ± 0.4	5.9 ± 1	<0.0001
**120' OGTT glucose [mg/dl]**	108.3 ± 19.3	164.4 ± 19.4	233.2 ± 21.7	<0.0001
Log Insulin sensitivity [min^-1^*10^-4^*mU/L]*	0.58 ± 0.19	0.38 ± 0.17	0.16 ± 0.16	<0.0001
**Free IGF-I [ng/ml]**	1.51 ± 1.13	1.20 ± 1.05	0.47 ± 0.56^†^	<0.0001
**HER-2 [ng/ml]**	9.6 ± 2.2	10.4 ± 1.8	11.4 ± 1.9^†^	<0.0001

Expressed as mean ± SD; *determined in 72 subjects with normal glucose tolerance, 26 with impaired glucose tolerance and 11 with type 2 diabetes.

Post-hoc analyses

^†^p < 0.0001 after Sheffé's test compared with subjects with normal glucose tolerance.

**Figure 2:**
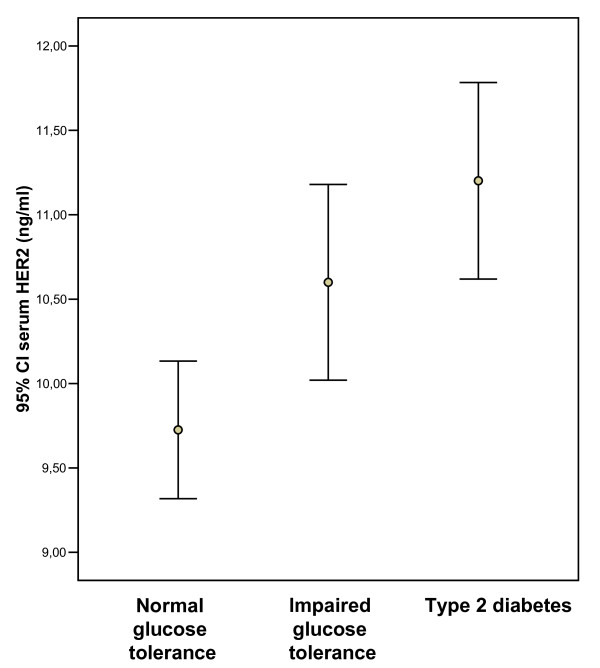
**95% confidence interval for the mean of serum HER-2 according to glucose tolerance status in subjects from the Cohort 1 study**.

Subjects with type 2 diabetes showed significantly decreased insulin sensitivity and significantly increased soluble serum HER-2 concentration (Table 1 and Figure 2).

In different multivariate regression models, fasting triglycerides emerged as the factor that independently contributed to 10-11% of serum HER-2 variance (Table 2).

**Table 2:** Multiple linear regression analyses with circulating HER-2 as dependent variable Serum glucose 120'OGTT, serum glucose at 120 mins of the oral glucose tolerance test

	*Dependent variable *CIRCULATING HER-2					
**Independent Variables**	**Beta**	**Sig**.	**Beta**	**Sig**.	**Beta**	**Sig**.
						
Age	0.03	0.7				
BMI	0.08	0.37	0.015	0.9	-0.017	0.87
Serum glucose at 120' OGTT			0.15	0.25		
Log insulin sensitivity	-0.19	0.057	-0.08	0.56	-0.20	0.09
Fasting triglycerides	**0.31**	**0.001**	**0.23**	**0.03**	**0.24**	**0.01**
***Adjusted R*^*2*^**	0.11		0.11		0.11	

### Cohort 2. Weight loss study

Characteristics of the subjects are shown in Table 3. Men and women were similar in age (45.2 ± 15.9 vs. 45 ± 14.5 years, p = 0.9) and BMI (37.2 ± 9.5 vs. 36 ± 9.8 kg/m^2^, p = 0.7). Interestingly, circulating HER-2 concentration was significantly increased in men (11.9 ± 1.9 vs. 8.5 ± 2.1 ng/ml, p < 0.0001) in parallel to insulin resistance (HOMA value 5.2 ± 3.2 vs. 2.18 ± 1.5 in men and women, respectively, p = 0.03). Serum HER-2 concentrations were significantly associated with fasting triglycerides and HOMA value in this cohort of obese subjects (Figure 1, right panels). Weight loss led to a significant decrease in serum HER-2 concentrations in parallel to changing anthropometric and metabolic parameters (Table 3). The decrease in circulating HER-2 was significant in both men (from 11.9 ± 1.9 to 9 ± 1.9 ng/ml, p = 0.001) and women (from 8.5 ± 2.1 to 7.5 ± 1.3 ng/ml, p = 0.03).

**Table 3:** Subjects' characteristics in the weight loss study

	Baseli[ne	Post-weight loss	
**Number of participants**	30 [15 men, 15 women]		
**Age [years]**	45.1 ± 14.9		
**BMI [kg/m^2^]**	36.6 ± 9.5	30.3 ± 5.4	**<0.0001**
**Percent body fat [%]**	43.7 ± 8.6	35 ± 9.6	**<0.0001**
**WHR**	0.96 ± 0.09	0.93 ± 0.07	**0.02**
**SBP [mmHg]**	128.2 ± 17.7	122 ± 14.9	0.1
**DBP [mmHg]**	81.8 ± 10.1	77.2 ± 7.5	**0.04**
**Fasting glucose [mg/dl]**	96 ± 11.9	89.8 ± 8.3	**0.01**
**Insulin [mU/l]**	18 ± 13	12 ± 6.1	**0.04**
**Total cholesterol [mg/dl]**	204.4 ± 35.7	175.6 ± 25.3	**<0.0001**
**HDL-cholesterol [mg/dl]**	53.2 ± 13.7	51.4 ± 12.8	0.3
**LDL-cholesterol [mg/dl]**	125.6 ± 32.8	108.6 ± 22	**0.009**
**Log 10 Fasting Triglycerides**	2.00 ± 0.17	1.87 ± 0.16	**<0.0001**
**HOMA-IR**	4.1 ± 3.4	2.5 ± 1.3	**0.04**
**HER-2 [ng/ml]**	10.2 ± 2.6	8.2 ± 1.8	**<0.0001**

**Figure 3:**
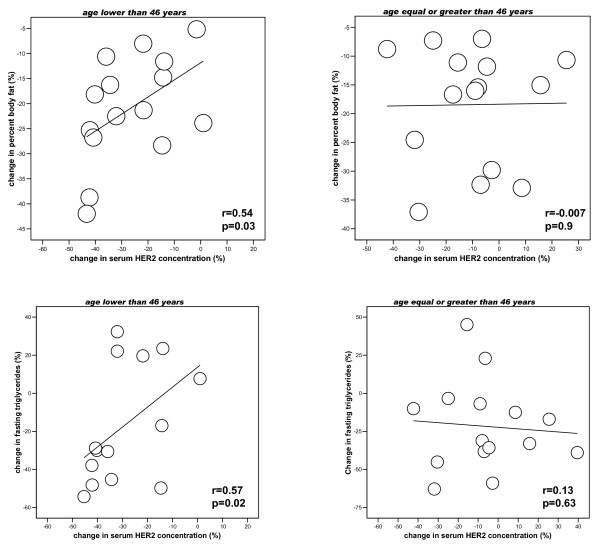
**Linear relationship between changing serum HER-2 concentrations, changing body fat [upper panels] and triglycerides [lower panels] according to the median age of the cohort**.

The change in serum HER-2 concentrations was not significantly associated with the change in BMI, percent body fat, insulin resistance or fasting triglycerides (r < 0.2, p > 0.05) in the whole cohort of subjects. However, when the subjects were divided according to the median age of the cohort (46 years), we found that changing serum HER-2 concentrations were significantly associated with the change in percent body fat and fasting triglycerides only in subjects aged less than 46 years (Figure 3).

## Discussion

Serum HER-2 concentrations are proportional to tissue HER-2 signalling activity [24]. We here describe that circulating HER-2 concentrations were positively associated with insulin resistance and fasting triglycerides in 2 cohorts of subjects. In different multiple linear regression analyses, fasting triglycerides constituted and independent factor that best explained the variance of circulating HER-2 in cohort 1 subjects. Furthermore, weight loss led to significantly decreased serum HER-2 concentrations. This decrease was especially remarkable in young subjects (arbitrarily defined as those below the median age of the cohort studied) in whom serum HER-2 concentration decreased from 11.1 ± 2.9 to 7.8 ± 1.7 ng/ml (p < 0.0001), while this decrease did not reach statistical significance in older subjects (9.77 ± 2.3 vs. 8.7 ± 1.6 ng/ml, p = 0.09). The decrease in young subjects was in parallel to decreased body fat and fasting triglycerides. The significance of this age-discordant effects remains to be established in a larger cohort of subjects. It should be recognized that the number of subjects studied is relatively small and the findings observed might be limited by study size. In cancer patients, the association between hormonal receptors and HER2/neu overexpression also varies with age. The hormone receptors are not an independent predictor for the HER2/neu status in young women while they are in elder patients [30].

A positive association between circulating concentrations of insulin or C-peptide and breast cancer risk has been observed in several [11-15], but not in all epidemiologic studies [16-18]. Type 2 diabetic patients in the present study showed both increased fasting insulin and serum HER-2 concentrations, two factors that may potentially increase cancer risk.

The mechanism behind the associations described here should be explored further. In this sense, it is important to note that Hsp90 regulates the intracellular trafficking and stability of both nascent HER-2 [31] and insulin and IGF-I receptors [19]. On the other hand, in patients with leprechaunism there are functional abnormalities of the EGF receptor, as well as of the insulin receptor, coined as multiple growth factor-resistant syndrome, that may contribute to the severity of the syndrome [32]. An interesting question raised by these studies was whether insulin resistance could lead to modulation of other receptors' function. In fact, evidence of EGF receptor abnormalities in several animal models of diabetes has been reported. Ob/ob and db/db mice were found to have a 70-80% decrease in EGF binding in liver membranes associated with decreased EGF receptor phosphorylation and decreased EGF receptor mRNA levels [33]. EGF binding and receptor phosphorylation was observed to be reduced in liver membranes from streptozotocin-treated diabetic rats [34]. The meaning of increased circulating HER-2 in a scenario of insulin resistance (current findings) should be explored further.

Metformin led to down-regulation of HER-2 in *in vitro *models [27], and overexpression of fatty acid synthase gene (implicated in the synthesis of endogenous fatty acids and triglycerides) activated HER1/HER2 tyrosine kinase receptors in human breast epithelial cells [35].

Regarding other potential mechanisms, cleavage of the HER2 ectodomain is a process that is inhibited by the tissue inhibitor of metalloproteases-1 [24]. Taking into account this information, we explored the association of circulating HER-2 with other circulating molecules whose concentrations are dependent on the activity of metalloproteases. Interestingly, in patients with type 2 diabetes, serum HER-2 concentrations were positively associated with serum soluble tumor necrosis factor-α (TNF-α) receptor 1 [r = 0.65, p = 0.001, n = 21] and serum DLK-1 (r = 0.81, p = 0.007, n = 9) (data not shown). In fact, TNF-α caused a dose-dependent decrease in insulin-stimulated IRS-1 phosphorylation and EGF-stimulated receptor autophosphorylation to 47-50% of control in in vitro studies. It could be speculated that TNF-α might be behind concomitant insulin resistance and EGF resistance, leading to increased circulating HER-2 levels [36].

Other members of the epidermal growth factor (EGF) family (neuregulins, which bind erb3 and erb4 receptors) modulate muscle metabolism by inducing glucose uptake, independently of insulin [37], and by regulating glucose transporter expression [38]. In fact, anti-neuregulin receptor-blocking antibodies impair contraction-induced glucose uptake [39]. Betacellulin-delta4 also ameliorates glucose intolerance in streptozotocin-treated rats [40].

The strenghts of this manuscript are the study of an homogenous sample of subjects and the use of a strong measure of insulin sensitivity (minimal model method) in cohort 1's subjects. Limitations include the evaluation of men in cohort 1 and of men and women in cohort 2.

In summary, circulating HER-2 concentrations were associated with insulin resistance in healthy subjects, significantly increased in patients with type 2 diabetes and decreased after weight loss in obese subjects. The role of serum HER-2 concentrations in the pathophysiology of insulin resistance and associated co-morbidities should be explored further.

## Competing interests

The authors declare that they have no competing interests.

## Authors' contributions

JMF-R participates in the analysis and interpretation of data and drafted the manuscript. JMM-N and AV-M carried out the biochemical analyses, participates in acquisition of data and in the biochemical and clinical determinations, and reviewed the manuscript. JAM and GF: Participate in design and reviewed the manuscript critically for important intellectual content. WR: Participate in design and coordination, and helped to draft the manuscript and reviewed the manuscript critically for important intellectual content. All authors read and approved the final manuscript.
